# Multipoint association mapping for longitudinal family data: an application to hypertension phenotypes

**DOI:** 10.1186/s12919-016-0049-2

**Published:** 2016-10-18

**Authors:** Yen-Feng Chiu, Chun-Yi Lee, Fang-Chi Hsu

**Affiliations:** 1 Institute of Population Health Sciences, National Health Research Institutes, Miaoli, 35053 Taiwan Republic of China; 2 Department of Biostatistical Sciences, Division of Public Health Sciences, Wake Forest School of Medicine, Winston-Salem, 27157 USA

## Abstract

It is essential to develop adequate statistical methods to fully utilize information from longitudinal family studies. We extend our previous multipoint linkage disequilibrium approach—simultaneously accounting for correlations between markers and repeat measurements within subjects, and the correlations between subjects in families—to detect loci relevant to disease through gene-based analysis. Estimates of disease loci and their genetic effects along with their 95 % confidence intervals (or significance levels) are reported. Four different phenotypes—ever having hypertension at 4 visits, incidence of hypertension, hypertension status at baseline only, and hypertension status at 4 visits—are studied using the proposed approach. The efficiency of estimates of disease locus positions (inverse of standard error) improves when using the phenotypes from 4 visits rather than using baseline only.

## Background

Approaches for analyzing longitudinal family data have been categorized into 2 groups [1]: (a) first summarizing repeated measurements into 1 statistic (eg, a mean or slope per subject) and then using the summarized statistic as a standard outcome for genetic analysis; or (b) simultaneous modeling of genetic and longitudinal parameters. In general, joint modeling is appealing because (a) all parameter estimates are mutually adjusted, and (b) within- and between-individual variability at the levels of gene markers, repeat measurements, and family characteristics are correctly accounted for [1].

The semiparametric linkage disequilibrium mapping for the hybrid family design we developed previously [2] uses all markers simultaneously to localize the disease locus without making an assumption about genetic mechanism, except that only 1 disease gene lies in the region under study. The advantages of this approach are (a) it does not require the specification of an underlying genetic model, so estimating the position of a disease locus and its standard error is robust to a wide variety of genetic mechanisms; (b) it provides estimates of disease locus positions, along with a confidence interval for further fine mapping; and (c) it uses linkage disequilibrium between markers to localize the disease locus, which may not have been typed. We extended this approach to map susceptibility genes using longitudinal nuclear family data with an application to hypertension. Four different outcomes were used based on the proposed method: (I) ever having hypertension (“Ever”), (II) incidence event with status changed from unaffected to affected (“Progression”), (III) first available visit as baseline only (“Baseline”), and (IV) all available time points (“Longitudinal”). We compared the estimates of the disease locus positions, their standard errors, the genetic effect estimate at the disease loci, and their significance for the 4 phenotypes to examine the efficiency gained from using repeated longitudinal phenotypes.

## Methods

### Genome-wide genotypes and phenotype data

Association mapping was conducted on chromosome 3 of the genome-wide association study (GWAS) data. A total of 65,519 single-nucleotide polymorphisms (SNPs) included in 1095 genes were genotyped on chromosome 3 for 959 individuals from 20 original pedigrees in Genetic Analysis Workshop 19 (GAW19). Of these individuals, there were 178 (38 %) affected offspring out of 469 offspring for phenotype (I) “Ever”; 130 (31 %) out of 421 offspring for phenotype (II) “Progression”; 64 (11 %) out of 600 offspring for phenotype (III) “Baseline”; and 60 (11 %) out of 565 offspring to approximately 85 (45 %) out of 189 offspring across the 4 visits (or 87 [21.63 %] out of 402 offspring on average) for phenotype (IV) “Longitudinal” (Table 1). To compare phenotypes (I) and (II), only individuals with at least 2 measurements were included in the “ever” phenotype. PedCut [3] was used to split large pedigrees with members more than 20 members into nuclear pedigrees. Consequently, we analyzed a total of 138 pedigrees with 1,495 individuals (the IDs for missing parents were added to form trios). In divided pedigrees, the nuclear families contained between 3 and 25 individuals. Five SNPs were removed because they failed the test of Hardy-Weinberg equilibrium (HWE) (*p* value < 10^−4^). The HWE test was performed using PLINK 1.07 [4] based on 56 unrelated subjects. (For information on PLINK, see http://pngu.mgh.harvard.edu/purcell/plink/.) A total of 22,056 genotypes from various SNPs with genotyping errors (genotyping error rate was around 3.51 × 10^−4^) were further excluded by the MERLIN 1.1.2 computing package (see http://www.sph.umich.edu/csg/abecasis/merlin/tour/linkage.html). None of the covariates was adjusted for in this approach.

**Table 1:** Number of offspring for different phenotypes

	Ever	Progression	Baseline	Visit 1	Visit 2	Visit 3	Visit 4
Affected offspring	178	130	64	60	78	125	85
All offspring	469	421	600	565	426	429	189
Percentage	0.38	0.31	0.11	0.11	0.18	0.29	0.45
Number of nuclear families	174	149	213	203	168	165	79

### Multipoint linkage disequilibrium mapping

Suppose M markers were genotyped in the region *R* at locations of 0 ≤ *t*_1_ < *t*_2_ < … < *t*_*M*_ ≤ *T*. We assume there are 2 alleles per marker. With *H* (*t*) being the target allele at marker position *t*, and *h* (*t*) being the nontarget allele, we define

$$ \begin{array}{l}{Y}_1^{D_{k_{il}}}(t)=\left\{\begin{array}{l}1\kern0.36em \mathrm{if}\;\mathrm{the}\;\mathrm{transmitted}\;\mathrm{paternal}\;\mathrm{allele}\;\mathrm{at}\;t\;\mathrm{is}\;H\;(t)\\ {}0\kern0.24em \mathrm{if}\;\mathrm{the}\;\mathrm{transmitted}\;\mathrm{paternal}\;\mathrm{allele}\;\mathrm{at}\;t\;\mathrm{is}\;h\;(t)\end{array}\right.,\;\\ {}{Y}_2^{D_{k_{il}}}(t)=\left\{\begin{array}{l}1\kern0.24em \mathrm{if}\;\mathrm{the}\;\mathrm{nontransmitted}\;\mathrm{paternal}\;\mathrm{allele}\;\mathrm{at}\;t\ \mathrm{is}\;H(t)\\ {}0\kern0.24em \mathrm{if}\;\mathrm{the}\;\mathrm{nontransmitted}\;\mathrm{paternal}\;\mathrm{allele}\;\mathrm{at}\;t\ \mathrm{is}\;h(t)\end{array}\right.,\end{array} $$ for the affected offspring ${D}_{k_{il}}$,

and $$ \begin{array}{l}{Y}_1^{{\overline{D}}_{k_{il}}}(t)=\left\{\begin{array}{l}\hbox{-} 1\kern0.36em \mathrm{if}\;\mathrm{the}\;\mathrm{transmitted}\;\mathrm{paternal}\;\mathrm{allele}\;\mathrm{at}\;t\;\mathrm{is}\;H\;(t)\\ {}0\kern0.24em \mathrm{if}\;\mathrm{the}\;\mathrm{transmitted}\;\mathrm{paternal}\;\mathrm{allele}\;\mathrm{at}\;t\;\mathrm{is}\;h\;(t)\end{array}\right.,\;\\ {}{Y}_2^{{\overline{D}}_{k_{il}}}(t)=\left\{\begin{array}{l}\hbox{-} 1\kern0.24em \mathrm{if}\;\mathrm{the}\;\mathrm{nontransmitted}\;\mathrm{paternal}\;\mathrm{allele}\;\mathrm{at}\;t\ \mathrm{is}\;H(t)\\ {}0\kern0.24em \mathrm{if}\;\mathrm{the}\;\mathrm{nontransmitted}\;\mathrm{paternal}\;\mathrm{allele}\;\mathrm{at}\;t\ \mathrm{is}\;h(t)\end{array}\right.,\end{array} $$ for the unaffected offspring ${\overline{D}}_{k_{il}}$. Then, we define the preferential transmission statistic ${Y}_{T_{k_{il}}}(t)={Y}_1^{D_{k_{il}}}(t)-{Y}_2^{D_{k_{il}}}(t)$ for the paternal side and ${X}_{T_{k_{il}}}(t)={X}_1^{D_{k_{il}}}(t)-{X}_2^{D_{k_{il}}}(t)$ for the maternal side for a trio; similarly, the preferential transmission statistic ${Y}_{U_{k_{il}}}(t)={Y}_1^{{\overline{D}}_{k_{il}}}(t)-{Y}_2^{{\overline{D}}_{k_{il}}}(t)$ and ${X}_{U_{k_{il}}}(t)={X}_1^{{\overline{D}}_{k_{il}}}(t)-{X}_2^{{\overline{D}}_{k_{il}}}(t)$ for an unaffected trio for both parental sides, respectively, where *k*_*il*_ = 1, …, *N*_1*il*_ (for unaffected), *N*_1*il*_ (*N*_2*il*_) is the number of affected (unaffected) offspring in the family *i* at the *l* ^th^ time point, *i* = 1, … *n*, *l* = 1, …, *L* (L = 1 or 4 in this study).

The expectation of the statistic is ${\mu}_{1\;{k}_{il}\;j\;}\left(\delta,\;\pi \right)=E\left[{Y}_{T_{k_{il}}}\left({t}_j\right)\;\left|{\varPhi}_1\right.\right]=\left(1-2{\theta}_{t_j,\;\tau}\right)\;C\;{\left(1-{\theta}_{t_j,\;\tau}\right)}^N\;{\pi}_j$ for case-parent trios and ${\mu}_{2\;{k}_{il}\;j\;}\left(\delta,\;\pi \right)=E\left[{Y}_{U_{k_{il}}}\left({t}_j\right)\;\left|{\varPhi}_2\right.\right]=\left(1-2{\theta}_{t_j,\;\tau}\right)\;C\;*{\left(1-{\theta}_{t_j,\;\tau}\right)}^N\;{\pi}_j$ for control-parent trios, where ${\theta}_{t_j,\;\tau }$ is the recombination fraction between marker position *t*_*j*_ and disease locus position *τ*, the recombination fraction Θ is a parametric function of the parameter of primary interest (*τ*, the physical position of the functional variant), *N* is the number of generations since the initiation of the disease variant, *Φ*_1_ denotes the event that the offspring is affected, *Φ*_2_ represents the event that the offspring is unaffected, $C=E\left[{Y}_{T_{k_{il}}}\left(\tau \right)\;\left|{\varPhi}_1\right.\right]=E\left[{X}_{T_{k_{il}}}\left(\tau \right)\;\left|{\varPhi}_1\right.\right]$, $C*=E\left[{Y}_{U_{k_{il}}}\left(\tau \right)\;\left|{\varPhi}_2\right.\right]=E\left[{X}_{U_{k_{il}}}\left(\tau \right)\;\left|{\varPhi}_2\right.\right],\delta =\left(\tau, N,C,C,*\right)$is the vector of parameters, and *π*_*j*_ = Pr [*h*(*t*_*j*_) |*h*(*τ*)]. ${\mu}_{1{k}_{il}j}$ is the probability for an affected offspring to receive a target allele, and $-{\mu}_{2{k}_{il}j}$ is the probability for an unaffected offspring to receive a target allele. The statistic ${Z}_{1{k}_{il}j}={X}_{T{k}_{il}j}+{Y}_{T{k}_{il}j}$ and ${Z}_{2{k}_{il}j}={X}_{U{k}_{il}j}+{Y}_{U{k}_{il}j}$ were used to estimate the parameters. The estimating equations used to solve for parameters *δ* are:

1 $$ {S}_1\left(\delta \right)={\displaystyle \sum_{i=1}^n{\displaystyle \sum_{l=1}^L{\displaystyle \sum_{k_{il=1}}^{N_{1il}}{\displaystyle \sum_{j=1}^M\frac{\partial {\mu}_{1{k}_{il}j\;}\left(\delta,\;{\widehat{\pi}}_j\right)}{\partial \delta }}}}}\;Co{v}^{-1}\left({Z}_{1{k}_{il}j}\right)\;\left\{{Z}_{1{k}_{il}j}-2{\mu}_{1{k}_{il}j}\;\left(\delta, {\widehat{\pi}}_j\right)\right\}, $$

2 $$ {S}_2\left(\delta \right)={\displaystyle \sum_{i=1}^n{\displaystyle \sum_{l=1}^L{\displaystyle \sum_{k_{il=1}}^{N_{2il}}{\displaystyle \sum_{j=1}^M\frac{\partial {\mu}_{2{k}_{il}j\;}\left(\delta,\;{\widehat{\pi}}_j\right)}{\partial \delta }}}}}\;Co{v}^{-1}\left({Z}_{2{k}_{il}j}\right)\;\left\{{Z}_{2{k}_{il}j}-2{\mu}_{2{k}_{il}j}\;\left(\delta, {\widehat{\pi}}_j\right)\right\}, $$

where ${\widehat{\pi}}_j$ is the average of nontransmitted parental alleles in the sample.

The estimating equations were solved iteratively for parameters *τ*, *N*, *C,* and C*, where *τ* and *C* are the 2 parameters of interest. The variance of the disease locus position estimate was estimated to make inferences about the disease locus position *(τ)* and its genetic effect *(C)* [2]. Theoretically, the genetic effect of *τ*, characterized by *C*, is the transmission probability that the affected offspring will carry the disease allele, *H*, at *τ*. Detailed derivations for case-parent trios in a cross-sectional design can be found in Chiu et al. [2, 5]. We will present the details of this proposed methodology elsewhere.

**Fig. 1:**
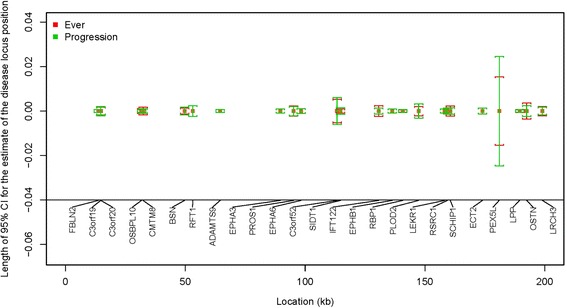
Length of 95 % confidence intervals (CIs) for the estimate of the disease locus position for “Ever” and “Progression” phenotypes

**Fig. 2:**
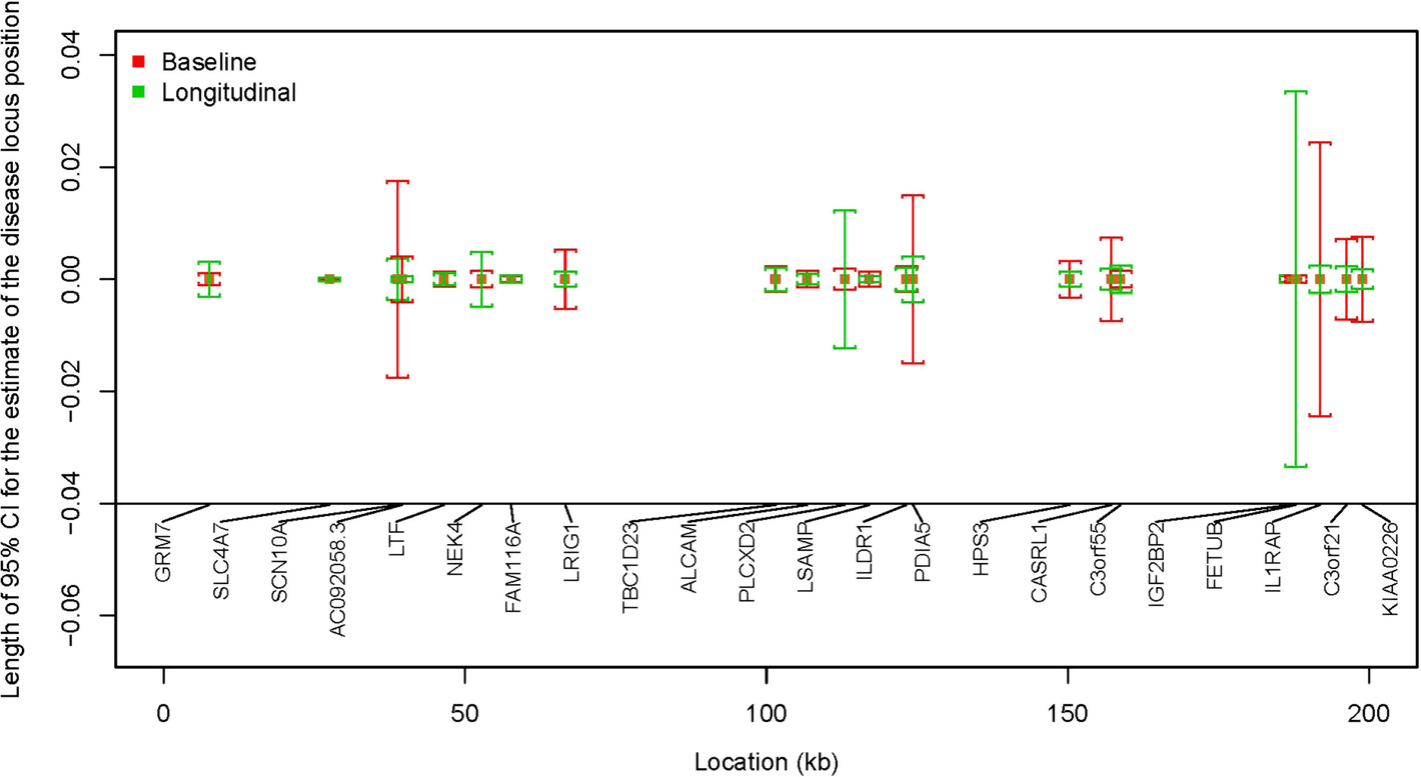
Length of 95 % confidence intervals (CIs) for the estimate of the disease locus position for “Baseline” and “Longitudinal” phenotypes

Gene-based association mapping was conducted for all SNPs on chromosome 3. This approach accounts for correlations between markers and repeated phenotypes within subjects, and correlations between subjects per family. The consistent estimates of hypertension locus position using “Ever” and “Progression” are shown in Table 2 and Fig. 1, while the consistent estimates of hypertension locus position using baseline and longitudinal data (at all 4 visits) are listed in Table 3 and Fig. 2.

**Table 2:** Significant and consistent estimates of disease locus positions and their genetic effects using “Ever” and “Progression” phenotypes

Gene*	Ever			Progression			Previous hits
	$\widehat{\tau}$ ± SE	*Ĉ*	*p* Value	$\widehat{\tau}$ ± SE	*Ĉ*	*p* Value	
*FBLN2*	13.6464 ± 0.00026	0.80	8.85 × 10^−7^	13.6462 ± 0.00030	0.88	2.47 × 10^−6^	L
*C3orf19*	14.6810 ± 0.00099	0.34	1.61 × 10^−12^	14.6802 ± 0.0010	0.34	6.87 × 10^−11^	L
*C3orf20*	14.7245 ± 0.00077	0.51	1.31 × 10^−6^	14.7244 ± 0.00091	0.45	2.81 × 10^−7^	L
*OSBPL10*	31.6853 ± 0.00051	0.41	7.69 × 10^−8^	31.6856 ± 0.00024	0.62	3.95 × 10^−6^	LG
*CMTM8*	32.3186 ± 0.00080	0.56	6.01 × 10^−6^	32.3183 ± 0.00052	0.70	1.20 × 10^−5^	
*BSN*	49.6596 ± 0.00062	0.83	3.42 × 10^−8^	49.6594 ± 0.00077	0.77	3.58 × 10^−6^	
*RFT1*	53.1117 ± 0.0012	0.37	4.07 × 10^−6^	53.1111 ± 0.0012	0.36	2.54 × 10^−5^	
*ADAMTS9*	64.5214 ± 0.00028	0.53	1.05 × 10^−11^	64.5216 ± 0.00030	0.54	2.54 × 10^−11^	L
*EPHA3*	89.6014 ± 0.00042	0.80	1.41 × 10^−6^	89.6018 ± 0.00040	0.89	1.19 × 10^−5^	
*EPHA6*	98.2999 ± 0.00047	0.41	3.57 × 10^−8^	98.2997 ± 0.00052	0.48	7.3 × 10^−7^	L
*C3orf52*	113.3097 ± 0.0026	0.62	6.55 × 10^−9^	113.3088 ± 0.0030	0.58	7.29 × 10^−6^	L
*SIDT1*	114.7743 ± 0.00039	0.78	8.45 × 10^−7^	114.7741 ± 0.00071	0.67	8.46 × 10^−6^	L
*IFT122*	130.7107 ± 0.0012	0.57	1.10 × 10^−5^	130.7118 ± 0.00060	0.71	4.90 × 10^−7^	
*RBP1*	140.7325 ± 0.00019	0.65	6.40 × 10^−7^	140.7345 ± 0.00033	0.42	3.89 × 10^−11^	L
*PLOD2*	147.3469 ± 0.00098	0.34	3.56 × 10^−6^	147.3469 ± 0.0016	0.34	3.02 × 10^−5^	L
*LEKR1*	158.2181 ± 0.00036	0.72	1.66 × 10^−10^	158.2183 ± 0.00043	0.77	4.35 × 10^−10^	L
*RSRC1*	159.4005 ± 0.00059	0.51	4.35 × 10^−6^	159.4003 ± 0.00064	0.51	1.64 × 10^−5^	L
*ECT2*	174.0021 ± 0.00064	0.88	1.91 × 10^−6^	174.0022 ± 0.00063	1.00	2.92 × 10^−7^	L
*PEX5L*	181.0080 ± 0.0078	0.29	2.99 × 10^−5^	181.0145 ± 0.013	0.23	5.23 × 10^−7^	LG
*LPP*	189.5573 ± 0.00035	0.50	1.71 × 10^−6^	189.5574 ± 0.00022	0.53	7.05 × 10^−6^	
*OSTN*	192.4272 ± 0.0018	0.73	5.33 × 10^−14^	192.4301 ± 0.0012	0.80	4.11 × 10^−9^	G

*Ĉ*, the genetic effect estimate; G, previous GWAS hits; L, previous linkage hits; $\widehat{\tau}$, the disease locus position estimate in cM

*Because of space limitations, we list only the 2 phenotypes with consistent estimates for the disease locus positions (the difference between the 2 $\widehat{\tau}$ for both phenotypes is less than 10^−2^ cM) and significant estimates for the genetic effects (both with *P* < 4.57 × 10^−5^, Bonferroni)

**Table 3:** Significant and consistent estimates of disease locus positions and their genetic effects using “Baseline” and “Longitudinal” phenotypes

Gene*	Baseline			Longitudinal			Previous hits
	$\widehat{\tau}$ ± SE	*Ĉ*	*p* Value	$\widehat{\tau}$ ± SE	*Ĉ*	*p* Value	
GRM7^†^	7.4917 ± 0.00048	0.44	2.87 × 10^−5^	7.4871 ± 0.0015	0.75	6.04 × 10^−14^	LG
*SLC4A7*	27.4521 ± 0.000045	0.30	0.014	27.4520 ± 0.000067	0.30	0.0024	LG
*SCN10A*	38.7559 ± 0.0089	0.088	0.019	38.7611 ± 0.0018	0.73	0.0022	
*AC092058.3*	39.5105 ± 0.0020	0.076	0.036	39.5102 ± 0.00024	0.21	0.00022	
*LTF*	46.4731 ± 0.00059	0.17	0.046	46.4733 ± 0.00045	0.31	0.0099	
*NEK4*	52.7326 ± 0.00071	0.83	0.00010	52.7277 ± 0.0024	0.86	0.00024	
*FAM116A*	57.6101 ± 0.00023	0.69	2.58 × 10^−6^	57.6107 ± 0.00032	0.61	0.011	
*LRIG1*	66.5968 ± 0.0026	0.28	0.018	66.5961 ± 0.00064	0.60	0.0022	L
*TBC1D23*	101.5084 ± 0.0011	0.46	0.026	101.5148 ± 0.0010	0.73	0.0011	L
*ALCAM*	106.7625 ± 0.00069	0.83	0.028	106.7598 ± 0.00041	0.62	0.00013	L
*PLCXD2*	112.9440 ± 0.00087	0.50	0.0016	112.9422 ± 0.0062	0.48	0.00020	L
*LSAMP*	117.0676 ± 0.00060	0.43	0.00022	117.0671 ± 0.00025	0.86	0.00012	L
*ILDR1*	123.2009 ± 0.0011	0.70	0.013	123.2008 ± 0.00098	0.91	0.023	
*PDIA5*	124.3194 ± 0.0076	0.065	0.0028	124.3225 ± 0.0020	0.68	0.0086	
*HPS3*	150.3484 ± 0.0016	0.14	1.65 × 10^−5^	150.3521 ± 0.00063	0.77	0.0080	L
*CASRL1*	157.2304 ± 0.0037	0.19	0.012	157.2295 ± 0.00094	0.28	0.031	L
*C3orf55*	158.7595 ± 0.00074	0.90	0.0051	158.7634 ± 0.0012	0.91	3.90 × 10^−6^	L
*IGF2BP2*	186.9725 ± 0.00018	0.74	0.041	186.9719 ± 0.00031	1.00	0.031	
*FETUB* ^*‡*^	187.8470 ± 0.00031	0.38	0.0012	187.8503 ± 0.017	0.042	0.0021	
*IL1RAP* ^*‡*^	191.8193 ± 0.012	0.074	0.0060	191.8203 ± 0.0012	0.79	4.75 × 10^−6^	
*C3orf21* ^*‡*^	196.2815 ± 0.0036	0.62	<10^−18^	196.2821 ± 0.0011	0.97	0.00057	
*KIAA0226*	198.9161 ± 0.0038	0.071	0.024	198.9168 ± 0.00076	0.15	0.022	

*Ĉ*, the genetic effect estimate; G, previous GWAS hits; L, previous linkage hits; $\widehat{\tau}$, the disease locus position estimate in cM

*Displayed are all genes where *p* ≤ 0.05

^†^The gene is significant with the Bonferroni correction (*P* < 4.57 × 10^−5^) and its *P* values are 2.31 × 10^−6^ and 0.00044 for “Ever” and “Progression,” respectively

^‡^ The same genes for the “Ever” and “Progression” phenotypes had *P* values <0.05 but > 4.57 × 10^−5^ for the genetic effect estimate

## Results and discussion

A total of 119 (11 %), 79 (7 %), 49 (4 %), and 42 (4 %) of 1095 genes had a significant genetic effect (*P* < 4.57 × 10^−5^ with Bonferroni correction) based on hypertension status at “Ever,” “Progression,” baseline (“Baseline”), and 4 visits (“Longitudinal”), respectively. There are only 3 significantly associated genes (*P* ≤ 0.05) for baseline and longitudinal phenotypes duplicated with the significantly associated genes for “Ever” and “Progression” outcomes: *FETUB, IL1RAP,* and *C3orf21*. Several hits identified here have been reported from linkage or GWAS studies. Table 2 shows genes with a significant genetic effect (*P* < 4.57 × 10^−5^). Table 3 presents the genes that are significant at a significance level of 0.05. Only 1 gene, *GRM7*, is significant at the level of *P* < 4.57 × 10^−5^.

Figures 1 and 2 display the 95 % confidence intervals for the estimate of the hypertension locus position for the 4 phenotypes centered at the estimated disease locus position. The comparison is shown for the genes listed in Tables 2 and 3. The standard errors of the estimates for the disease locus position are smaller in 64 % of the genes based on longitudinal data (Table 3) compared to those based on baseline data. This is because those incidence cases included in “Progression” were also included in the analysis of “Ever.” Only prevalent cases, a relatively small proportion, are additionally included in the analysis of “Ever.” Thus, the results from “Progression” and “Ever” are similar.

## Conclusions

Methods of genetic analysis rely heavily on correlations among family members’ outcomes to infer genetic effects, whereas longitudinal studies allow investigators to study factors’ effects on outcomes and changes over time [1]. To retrieve full information from longitudinal family data, appropriate statistical approaches are crucial. We proposed a multipoint linkage disequilibrium approach accounting for multilevel correlations between markers per subject, within-subject longitudinal observations, and subjects within families, aiming to correctly localize the disease locus and assess its genetic effects. This approach has several advantages: it allows us to estimate the disease locus position, the disease locus’s genetic effect, and the 95 % confidence intervals without specifying a disease genetic mode and yet making full use of the markers and repeated measurements. In addition, this approach treats the genotype data as random conditional on the phenotype, eliminating the problem of ascertainment bias. We applied this approach to the baseline and longitudinal prevalence/incidence of hypertension events. The efficiency of parameter estimates was similar for the “Ever” and “Progression” categories, but was improved with repeated longitudinal outcomes compared to the use of “Baseline” only. This difference between analyses might largely result from the different total sample sizes and proportions of hypertensive subjects for different phenotypes. Several identified genes on chromosome 3 for hypertension were consistent with findings from previous linkage and association studies. Despite its advantages, this proposed approach also has limitations; for example, covariate adjustment is not available.
